# A genome-wide association study identifies candidate genes for target leaf spot disease resistance in adult cucumber (*Cucumis sativus* L.)

**DOI:** 10.3389/fpls.2025.1542274

**Published:** 2025-06-06

**Authors:** Ying’ao Xu, Shaoyun Dong, Xuewen Xie, Diane M. Beckles, Xiaoping Liu, Jiantao Guan, Caixia Li, Xingfang Gu, Han Miao, Shengping Zhang

**Affiliations:** ^1^ State Key Laboratory of Vegetable Biobreeding, Institute of Vegetables and Flowers, Chinese Academy of Agricultural Sciences, Beijing, China; ^2^ Department of Plant Sciences, University of California, Davis, Davis, CA, United States

**Keywords:** cucumber, target leaf spot, disease resistance, GWAS, candidate gene analysis

## Abstract

Target leaf spot disease (TLS), caused by *Corynespora cassiicola* (Berk & Curt) Wei, ranks among the most serious fungal diseases affecting cucumber production. However, the genetic basis for TLS resistance in cucumber has not yet been determined. In this study, we evaluated TLS resistance in the adult plants of 130 cucumber accessions using a disease index (DI) in October 2021, June 2023, and October 2023. The accessions used in this study were representative collection selected from the global 3,342 accessions, and contain four ecotypes (the Eurasian, Indian, East Asian, and Xishuangbanna type). Cluster analysis suggested that 11 of the 130 accessions exhibited high levels of TLS resistance (CG28, CG70, CG84, CG86, CG100, CG104, CG98, R163, R61, CG64, CG71). A genome-wide association study (GWAS) analysis was then performed based on the BLUP value of the DI collected from these three seasons, and three loci (*gTLS5.1*, *gTLS5.2*, and *gTLS7.1*) associated with TLS on two chromosomes were identified. Seven candidate genes linked to disease resistance and abiotic stress were identified through functional annotation with Arabidopsis orthologous genes and pairwise linkage disequilibrium (LD) correlation analysis. Sequence alignment, expression and haplotype analysis further indicated to five of these candidate genes as being potentially causal to TLS: *CsaV3_5G010580* for *gTLS5.1*, and *CsaV3_7G026140, CsaV3_7G026180*, *CsaV3_7G026200* and *CsaV3_7G026220* for *gTLS7.1*. These genes related to TLS resistance in cucumber, could be useful to promote cucumber breeding and development.

## 1 Introduction

Cucumber (*Cucumis sativus* L.) is a commercially significant vegetable crop cultivated globally. According to the FAOSTAT (http://www.fao.org/cucumber-statistics), the area under cucumber cultivation in China has reached 1.31 million hectares, with a total production of 77.3 million tons in 2022. Target leaf spot disease (TLS) caused mainly by the fungus *Corynespora cassiicola* (Berk & Curt) Wei, is one of the most important diseases in cucumber and occurs extensively in many countries in differencing geographical regions. In China, TLS was first detected as a cucumber disease in Jiangxi Province in 1960. Since 1992, outbreaks of cucumber TLS have occurred in Liaoning, and in recent years it has developed into one of the most common and serious cucumber diseases. Currently, the disease has been observed widely throughout China, being prominent in Northern and Northeastern China, reducing cucumber yields by 20%-70% in severe cases (Li et al., 2008; Yang et al., 2012).

TLS is a foliar disease that can occur throughout the entire growing seasons, but typically appears during the middle and late growth stages of cucumber cultivation. The development of symptoms is highly susceptible to environmental factors. Warm and humid conditions are more likely to result in the disease occurrence because the spores require moisture to germinate and initiate new infections (Zou et al, 2002). TLS develops first on the middle and lower leaves and then spreads to the upper leaves (Yu et al., 2014). At the early stage of the disease, small yellow-brown water-soaked spots approximately 1 mm in diameter emerge on the leaves. As the disease progresses, the lesions expand into a rough, even can grow to 10–15 mm, becoming round or irregular in shape (Li, 2010). The lesion is brown, with a gray-white, translucent center. In severe cases, multiple lesions merge into large spots, covering more than 95% of the leaf area, potentially leading to leaf death (Yang et al., 2012). Infected leaves lose their photosynthetic function, which reduces the yield of TLS-infected plants, and consequently affects the economic income of farmers. Therefore, research on the molecular mechanisms of TLS resistance and the breeding of TLS-resistant varieties in cucumbers are urgent problems to be solved.

Few studies on the inheritance and QTL mapping for TLS resistance have been reported in cucumber. Abul-Hayja et al. (1978) reported that a dominant single gene, i.e., *Cca*, controls cucumber TLS resistance, while other studies suggest that cucumber TLS resistance is controlled by recessive genes. Wang et al. (2010) discovered that cucumber TLS resistance was governed by a single recessive gene (*cca-1*) linked to marker CSFR33 in the resistant Q5 parent. Yang et al. (2012) reported the discovery of a recessive allele (*cca-2*) that controls TLS resistance in the wild cucumber accession PI 183967 and localized *cca-2* between markers SSR10954 and SSR16890 on Chromosome 6. They speculated that *cca-1* and *cca-2* are likely to be the same gene. Wen et al. (2015) used an F_2:3_ population derived from the resistant line D31 to fine-map a cucumber TLS resistance locus, naming it *cca-3*. The *cca-3* locus is located within a 79-kb region on Chromosome 6, and they identified a potential candidate gene, *Csa6M375730*, which encodes a *CC-NB-ARC* type resistance homolog. The discovery of these disease-resistance genes provides a foundation for developing molecular markers for breeding and understanding the genetic mechanisms underlying disease resistance.

A genome-wide association study (GWAS) is a statistical approach for mapping quantitative trait loci. The entire genome of hundreds of individuals is screened for polymorphisms linked to phenotypes of interest, taking advantage of historical linkage disequilibrium. GWAS has become a routine strategy for deciphering the associations between genotype and phenotype in a wide range of species (Liu and Yan, 2019), including for disease resistance. A GWAS analysis by Wu et al. (2020) identified 33 SNPs associated with powdery mildew (PM) resistance in 117 bottle gourd accessions, highlighting the disease-resistant gene *HG_GLEAN_10015218* on chromosome 2. Liu et al. (2020) detected 18 loci associated with downy mildew (DM) resistance in cucumber, two of which were novel, i.e., *dmG2.1* and *dmG7.1*. Li et al. (2021) conducted a GWAS analysis of 89 bottle gourd accessions, identifying ten SNPs significantly associated with *fusarium* wilt resistance, and identifying *HG_GLEAN_10011803* as a candidate gene. Han et al. (2023) inoculated cucumber with the pathogen causing gummy stem blight (GSB) disease and performed a GWAS analysis. They identified 35 SNPs associated with GSB resistance and, among them, four candidate genes, i.e., *Csa3G129470*, *Csa5G606820*, *Csa5G606850*, and *Csa6G079730*. To identify candidate genes regulating bacterial soft rot (BSR) resistance in cucumber, Zhang et al. (2024) investigated four loci: *gBSR2.1*, *gBSR2.2*, *gBSR3.1*, *gBSR4.1* and *gBSR5.1* identified through a GWAS. These studies show the effectiveness of GWAS for mining resistance genes and elucidating the underlying molecular mechanisms in related studies.

In this study, we evaluated TLS resistance in cucumber at the adult stage using 130 accessions. GWAS analysis was then performed based on the BLUP value of the TLS data collected from three seasons. Candidate genes associated with TLS resistance were predicted based on functional annotation, sequence alignment, and expression analysis. Our study identified key loci associated with TLS resistance in cucumber, facilitating a detailed analysis of their functions and mechanisms, and laying the foundation for accelerated breeding of resistant varieties.

## 2 Materials and methods

### 2.1 Plant material

A panel of 130 cucumber accessions was provided by the cucumber research group at the Institute of Vegetables and Flowers, at the Chinese Academy of Agricultural Sciences in China (**Supplementary Table S1**). These accessions, representing four distinct ecotypes (the Eurasian, Indian, East Asian, and Xishuangbanna type), were selected from a global collection of 3,342 cucumber germplasms based on geographic adaptation and phenotypic diversity (Turesson, 1922; Huang et al., 2009; Qi et al., 2013). The specific categories are listed in **Supplementary Table S2**. All accessions were planted in Shouguang, Shandong Province, and all experiments were conducted in a randomized complete block design (RCBD), with three replications, and five plants per replication, with high phenotypic consistency across replicates.

### 2.2 Inoculation and phenotypic analysis of TLS

**Figure 1:**
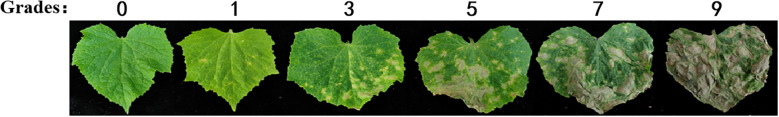
The rating scale of TLS resistance. The numbers above the top represent the TLS grade.

Adult plants were phenotyped for TLS resistance in October 2021, June 2023, and October 2023. Naturally TLS infection occurred at the adult plant stage, TLS symptoms appeared approximately six weeks after sowing. TLS resistance was evaluated weekly for three consecutive weeks, commencing seven weeks after sowing. The disease severity of each plant was assessed using the criteria outlined by Kan et al. (2007), a widely recognized and standardized method in the field, which has been adopted in studies, such as those by Yang et al. (2012) and Wen et al. (2015). Each plant was ranked on a disease grade of 0, 1, 3, 5, 7, and 9 which describes the percentage of the average TLS lesion area of the 10th to 15th functional leaves (the first true leaf count as the first leaf) of each plant: manifesting the disease symptoms as follows: Grade 0: = 0%; Grade 1: ≤ 5%; Grade 3: 5 - 25%; Grade 5: 25-50%; Grade 7: 50-75%; Grade 9: ≥75% of the leaf area (**Figure 1**).

*Corynespora cassiicola* (Berk & Curt) Wei used for inoculation for qPCR was provided by the vegetable disease prevention and control innovation team (Institute of Vegetables and Flowers, Chinese Academy of Agricultural Sciences). Incubate the preserved *Cca* on potato dextrose agar (PDA) plates at 28°C for 7 days to allow sufficient spore production. Then add 4 ml sterile water per petri dish and use a sterilized brush to scrape the spores of the cucumber leaf spot pathogen down, preparing a spore suspension with a concentration of 1× 10^5^ spores· mL ^-1^ for inoculation (Kan et al., 2007; Zou et al., 2002).

For each accession, the disease index (DI) was calculated according to the following formula:


$$\text{DI }\left(\%\right)=100\times \frac{\sum (\text{Number of plants with disease rating }\times \text{Disease rating})}{\text{Highest disease rating }\times \text{Total number of plants}}$$

For each experiment, the DI of each line was calculated by taking the average of the DI in three replicates, and the best linear unbiased prediction (BLUP) values of the DI obtained from the three seasons were used for GWAS analysis.

### 2.3 Methods of statistical analysis

The analysis of variance was performed on the obtained DI values using IBM SPSS Statistics 20 software (Manickam et al., 2023). In the R software, the Pearson correlation coefficient was used for correlation analysis, the ‘ggtree’ package was utilized for cluster analysis and the Best Linear Unbiased Predictor (BLUP) was calculated using the ‘lme4’ package (Team, R.C, 2014). BLUP was selected as the optimal method for analysis because it can reduce the influence of environmental factors giving more accurate and reliable results (VanRaden, 2008).

### 2.4 Genome-wide association analyses and linkage-disequilibrium analysis of TLS

The genomes of the 130 cucumber accessions have been sequenced and are available in the NCBI database (PRJNA171718 and PRJNA831637). A total of 1,642,918 high-quality SNPs were used for the GWAS in this study. These SNPs were uniformly distributed across the seven chromosomes of the cucumber genome. A factorial spectral transformed linear mixed model (FaST-LMM) was employed for association tests of TLS resistance with estimated kinship matrices as covariates, with a threshold of 5.1 (Lippert et al., 2011; Jiang et al., 2021). The PLINK software was employed to calculate the linkage disequilibrium (LD) decay coefficient (r²) among high-quality SNPs and evaluate LD decay following established methods (Liu et al., 2020). LD blocks were defined using a threshold of r²≥ 0.6 (Li et al., 2023), with pairwise LD relationships visualized through a heatmap generated by the LD heatmap package (Shin et al., 2006).

### 2.5 Candidate gene analysis

Firstly, the 50-kb intervals upstream and downstream of phenotype-associated SNP peaks were selected as the regions to screen for candidate genes (Purcell et al., 2007). Secondly, candidate genes were annotated using the sequence of the *Cucumis sativus* L. var. sativus 9930 version 3 genome (http://cucurbitgenomics.org/organism/20), and then were further analyzed by haplotype analysis. Thirdly, candidate genes associated with disease resistance and abiotic stress were selected according to their predicted functionality using TAIR (http://www.arabidopsis.org/index.jsp), Swiss-Prot (http://www.uniprot.org/), and the Gene Ontology (GO) database (http://amigo.geneontology.org/amigo/landing). Finally, candidate genes were identified based on their expression levels as determined by real-time quantitative polymerase chain reaction (qRT-PCR).

### 2.6 RNA extraction and qRT-PCR verification

Four TLS-resistant accessions (‘CG70’, ‘CG71’, ‘CG72’ and ‘CG78’) and four TLS-sensitive accessions (‘CG107’, ‘R16’, ‘R28’ and ‘R31’) were selected for qRT-PCR. The first true leaf was collected at 0h, 12h, and 24h post-inoculation and stored at -80°C. Three biological replicates and three technical replicates were established for each treatment. Total RNA was extracted from samples using the RNeasy Plant Mini Kit (TaKaRa 9769, Takara Bio, Inc. Otsu, Japan). The quantitative real-time polymerase chain reaction (qRT-PCR) was conducted using the SYBR Premix Ex TaqTM II (TaKaRa, Takara Bio, Inc., Otsu, Japan. *Actin1* (*Csa3G806800*) was employed as the reference gene (Xie et al., 2018). The expression level of *Actin1* was set as a control for normalization, to evaluate the relative expression values of the candidate genes using the 2^−ΔΔCt^ method (Jana and Jiban, 2010). The specific primers for each gene are listed in **Supplementary Table S3**.

## 3 Results

### 3.1 Genetic diversity analysis of TLS resistance in cucumber

**Figure 2:**
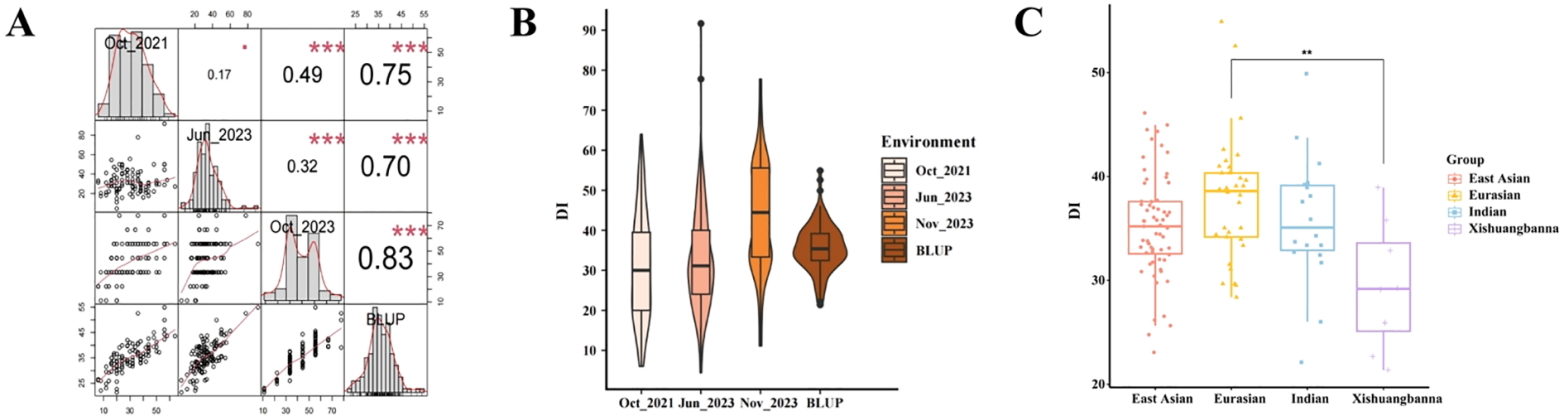
Violin and box plots showing the phenotypic and ecotype distribution of TLS in 130 cucumber accessions. **(A)** Correlation analysis of TLS in 130 cucumber accessions evaluated in Oct_2021, Jun_2023, and Oct_2023 and their BLUP analysis. Pearson correlation and frequency distribution of the disease index (DI). *** indicates significance at *p*< 0.001, * indicates significance at *p*< 0.05. **(B)** Violin plots illustrate the phenotypic distribution of the DI in three different environments. **(C)** Box plots show the distribution of the BLUP values for the four ecotypes.** indicates significance at *p*< 0.01.

A total of 130 cucumber accessions were evaluated for their TLS resistance in October 2021, June 2023, and October 2023, respectively. The DI value was calculated for each accession in each experiment based on the size, i.e., area of the TLS lesion. The coefficient of variation (*CV*) for the data collected in 2021 was 71.40%, with a mean DI value of 24.28. The *CV* for June 2023 was 42.91%, and for October 2023 it was 47.73%, with a mean DI value of 31.86 and 38.80, respectively (**Supplementary Table S4**). These data validate that the accessions we used have significant genetic variation. Furthermore, the DI values from the three seasons were highly correlated (**Figure 2A**). The distribution of DI values is depicted in a violin plot (**Figure 2B**), suggesting that TLS resistance in cucumber is a quantitative trait with a normal distribution, and multiple genes influence the inheritance of TLS resistance in cucumber. BLUP was selected as the optimal method for analysis.

The 130 cucumber accessions comprised of the Xishuangbanna (n=9 lines), the Indian (n=18 lines), the East Asian (n=63 lines), and the Eurasian types (n=32 lines). The BLUP values of the Xishuangbanna type were significantly lower than those of the other three ecotypes, so the Xishuangbanna type was considered to be more resistant to TLS, while the Eurasian type which had higher values, was more susceptible to TLS (**Figure 2C**).

### 3.2 Cluster analysis of TLS resistance in cucumber germplasms

**Figure 3:**
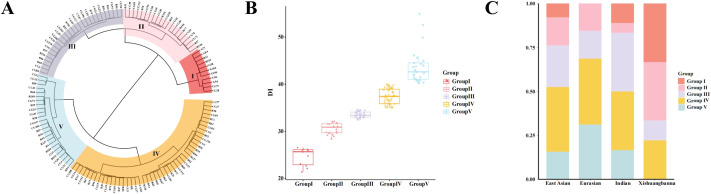
Classification of TLS resistance across cucumber accessions based on the analysis of BLUP values Disease Indices (DI). **(A)** Group analysis of the BLUP values for accessions. The DI response was grouped into five categories - I, II, III, IV, and V, with Group I being the most resistant and V the most susceptible to TLS. **(B)** The range of the BLUP values from Group I to Group V. **(C)** The proportion of accessions derived from the four ecotypes, distributed across Groups I-V.

Using their BLUP values, the 130 accessions were categorized into five groups using the Ward linkage method (**Figure 3A**). The classification was as follows: (a) Group I (very highly TLS-resistant accessions, n=11 lines), (b) Group II (highly TLS-resistant accessions, n=21 lines), (c) Group III (medium TLS-resistant accessions, n=27 lines), (d) Group IV (highly TLS-sensitive accessions, n=46 lines), and (e) Group V (very highly TLS-sensitive accessions, n=25). These five groups exhibit significant differences, with the DI gradually increasing across these categories (**Figure 3B**). These five groups also differ in their distribution and proportion of the four ecotypes, and each ecotype included accessions from multiple clusters (**Figure 3C**). The East Asian and Indian ecotypes were distributed across all five groups, whereas no Eurasian lines were in Group I, and no Xishuangbanna lines were in Group V (**Figure 3C**). The Xishuangbanna ecotype had the highest proportion of accessions in Groups I and II, suggesting that the Xishuangbanna ecotype exhibits stronger disease resistance compared to the other three ecotypes.

### 3.3 Genome-wide association analysis of TLS resistance in adult cucumber plants

**Figure 4:**
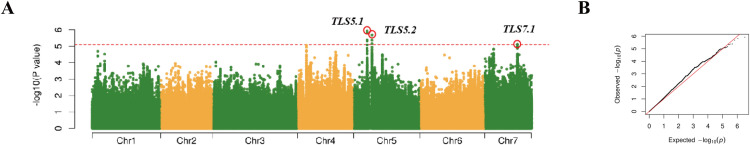
GWAS Manhattan plot **(A)** and Q-Q plot **(B)** of TLS resistance based on BLUP values. The red horizontal line represents a significance threshold of 5.1 (-log_10_(*p*) > 5.1), and the strongest peaks are marked with a red circle.

Phenotypic data were collected from plants grown in three different environments in Shouguang, Shandong in 2021 and 2023, respectively, and the GWAS plots for each season have been included in the appendix (**Supplementary Figure S1**). Comprehensive details of all GWAS loci are summarized in **Table 1**. The BLUP values derived from data collected in these three seasons were used for genome-wide association analysis (Ma et al., 2021). The GWAS results demonstrated the presence of three loci (*gTLS5.1*, *gTLS5.2*, and *gTLS7.1*) with a threshold of 5.1 across two chromosomes (**Figure 4**).

**Table 1:** The highly significant SNPs at the *gTLS5.1*, *gTLS5.2* and *gTLS7.1* loci by GWAS.

Environment	GWAS signal sites	Physical position (bp)	Interval (bp)	-log_10_ (*P*)
	*gTLS5.1*	6,604,733	6,554,733-6,654,733	5.92
Jun_2023SG	*gTLS5.2*	8,963,257	8,913,257-9,013,257	5.69
	*gTLS7.1*	15,651,307	15,601,307-15,701,307	5.12

### 3.4 Analysis of the identified loci for candidate genes

We further explored the *gTLS5.1*, *gTLS5.2* and *gTLS7.1* loci identified by GWAS, to identify reliable candidate genes. We used the 50-kb intervals upstream and downstream of the peak SNPs for further analysis, and the genes contained in each locus are shown in **Supplementary Table S5**. Genes within this interval were screened by functional annotation using *Arabidopsis* orthologues. A combination of haplotype and sequence analysis, and qRT-PCR, was then used to further narrow the list of candidate genes. For gene expression analysis, four resistant accessions (‘CG70’, ‘CG71’, ‘CG72’ and ‘CG78’) and four sensitive accessions (‘CG107’, ‘R16’, ‘R28’ and ‘R31’) were selected for analysis. Through the aforementioned analyses, five candidate genes, i.e., *CsaV3_5G010580*, *CsaV3_7G026140*, *CsaV3_7G026180*, *CsaV3_7G026200*, and *CsaV3_7G026220* which appear to be associated with TLS resistance were preliminarily predicted.

**Figure 5:**
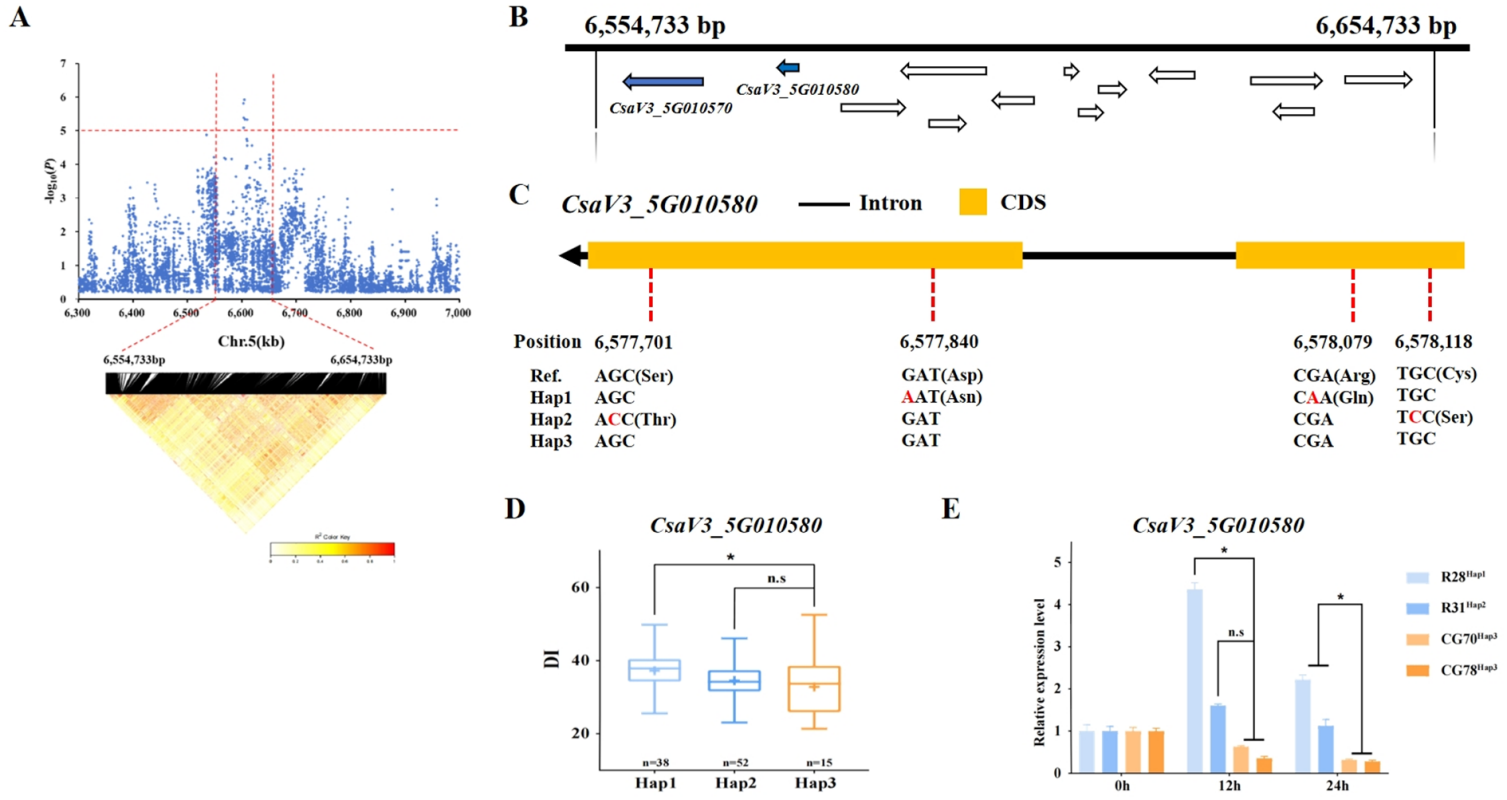
Candidate gene analysis of the *gTLS5.1* locus. **(A)** Local Manhattan plot (above) and LD heatmap (below) surrounding the locus. The red horizontal dashed line indicates the significance threshold (-log_10_(*P*) > 5.1). The vertical red dashed lines indicate the candidate region (100 kb). **(B)** Thirteen genes are depicted with arrows showing their direction in the *gTLS5.1* candidate region. Blue arrows indicate genes linked to abiotic stress or disease resistance, with detailed annotations in **Supplementary Table S6**. **(C)** The structure of *CsaV3_5G010580*. The orange rectangles represent the CDS, the black horizontal line represents introns, and ‘Ref.’ represents the sequence in the syntenic region of reference cucumber genome (Chinese Long v3). **(D)** Box plots showing a haplotype analysis of *CsaV3_5G010580* with *Hap*1, *Hap2*, and *Hap3* genotypes. Blue boxes represent the DI of TLS-sensitive accessions carrying *Hap1* (light blue) and *Hap2* (dark blue) alleles. Orange boxes represent the DI of TLS-resistant accessions carrying *Hap3* (* *p<* 0.05). **(E)** The expression level of *CsaV3_5G010580* in the *Hap1* (‘R28’), *Hap2* (‘R31’), and *Hap3* genotypes (‘CG70’ and ‘CG78’) at 0, 12, and 24 h post-inoculation treatment. *Actin1* was used as an internal control. Data are represented as average values with the mean ± standard deviation (SD) of three independent biological replicates. Significant difference (* *p<* 0.05). n.s. indicates non-significant differences (p ≥ 0.05).

For *gTLS5.1*, the 100 kb (6,554,733 - 6,654,733 bp) region on chromosome 5 (Chr.5) was analyzed using pairwise LD correlations (**Figure 5A**). According to the reference cucumber genome (v3), 13 annotated genes are contained in this region. Among them, *CsaV3_5G010570* encodes an SNF2 domain-containing protein, CLASSY 1-like. The CLASSY family controls DNA methylation in *Arabidopsis* (Zhou et al., 2022). However, no SNPs were detected in this gene.

Another gene *CsaV3_5G010580* encoding a SPX (SYG1/Pho81/XPR1) domain-containing protein/zinc finger (C3HC4-type RING finger) related-protein, may be associated with disease and stress resistance in plants (**Supplementary Table S6**). Four SNPs, which would lead to non-synonymous mutations in the coding sequence (CDS) region were identified in *CsaV3_5G010580* (**Figure 5C**). The amino acid changes of ‘R28’ carrying *Hap1* were Asp → Asn and Arg → Gln at the corresponding positions. Ser → Thr and Cys → Ser were changed in the CDS of ‘R31’ carrying *Hap2*. And these SNPs may change protein function. Genotyping of the 130 accessions revealed that 38 belonged to the *Hap1* type, 52 to the *Hap2* type, and 15 to the *Hap3* type. A comparison of the DI values of the three haplotypes revealed a difference between *Hap1* and *Hap3*, but no significant difference between *Hap2* and *Hap3* (**Figure 5D**). *Hap1* and *Hap2* showed sensitivity to TLS, whereas *Hap3* showed more resistance. The relative expression of *CsaV3_5G010580* was lower in *Hap3* (‘CG70’ and ‘CG78’), but significantly higher in *Hap1* (‘R28’) and *Hap2* (‘R31’) at 12 hours post-inoculation (**Figure 5E**). The expression level of *Hap1* (‘R28’) and *Hap2* (‘R31’) then gradually decreased, while that of *Hap3* (‘CG70’ and ‘CG78’) continued to show a decreasing trend (**Figure 5E**).

At the *gTLS5.2* locus, the 100 kb (8,913,257 - 9,013,257 bp) region on Chr.5 was analyzed using pairwise LD correlations (**Supplementary Figure S3**). By examining the syntenic region of the reference cucumber genome (Chinese Long v3), three annotated genes were identified (**Supplementary Table S5**). Only one, i.e., *CsaV3_5G012840* was involved in regulating abiotic stresses, but there were no SNPs in this gene.

**Figure 6:**
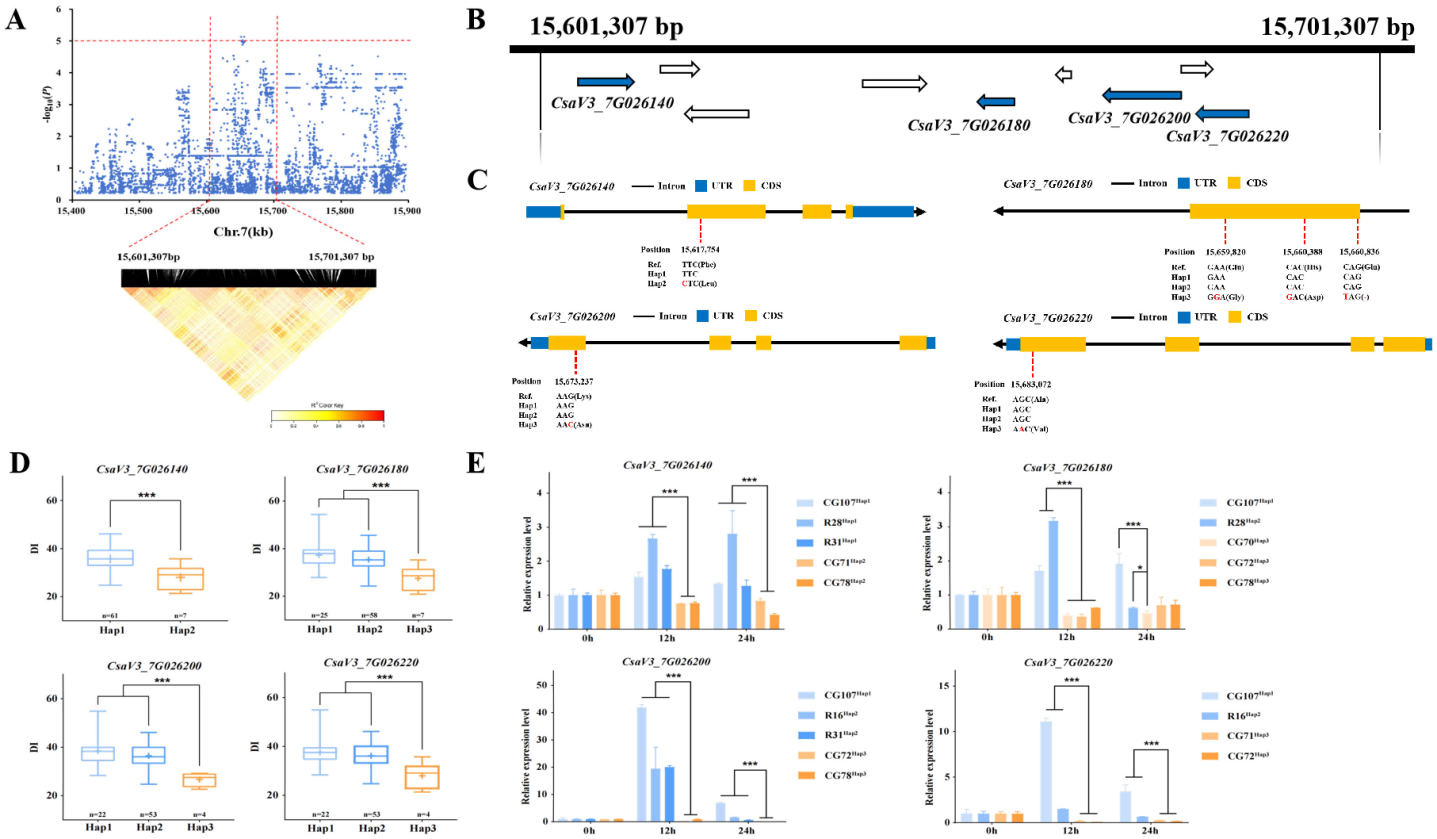
Candidate gene analysis of the *gTLS7.1* locus. **(A)** Local Manhattan plot (above) and LD heatmap (below) surrounding the locus. The red horizontal dashed line indicates the significance threshold (-log_10_(*P*) > 5.1), and the vertical red dashed lines indicate the candidate region (100 kb). **(B)** Nine genes are depicted with arrows showing their polarity. The blue arrows indicate genes linked to abiotic stress or disease resistance, with detailed annotations in **Supplementary Table S6**. **(C)** The structures of *CsaV3_7G026140*, *CsaV3_7G026180*, *CsaV3_7G026200*, and *CsaV3_7G026220*. The blue and orange rectangles represent the UTRs and CDSs respectively, and the black horizontal line represents introns. **(D)** Box plots showing the haplotype analysis of *CsaV3_7G026140* with *Hap1* and *Hap2* lines, while showing haplotype analysis of *CsaV3_7G026180*, *CsaV3_7G026200* and *CsaV3_7G026220* with *Hap1*, *Hap2* and *Hap3* genotypes. The blue boxes represent the DI of the TLS-sensitive accessions carrying *Hap1* (light blue) and *Hap2 (darker blue)*. The orange box represents the DI of TLS-resistant accessions carrying *Hap3* (*** *p*< 0.001). **(E)** The expression level of *CsaV3_7G026140*, *CsaV3_7G026180*, *CsaV3_7G026200*, and *CsaV3_7G026220* at 0, 12, and 24 h post-inoculation treatment. *Actin1* was used as an internal control. Data are the mean values with the SD of three independent biological replicates. Significant difference (*** *p<* 0.001, * *p<* 0.05). n.s. indicates non-significant differences (p ≥ 0.05).

The 100 kb region flanking the *gTLS7.1* locus, spanning from 15,601,307 - 15,701,307 bp on Chr.7 was analyzed using pairwise LD correlations (**Figure 6A**). Nine annotated genes were predicted to be in this region (**Supplementary Table S5**). *CsaV3_7G026140* encodes RPM1-interacting protein 4, which plays a role in plant immunity. *CsaV3_7G026180* encodes a phenolic glucoside malonyltransferase, which plays a role in mediating cold and salt stress signaling in *Arabidopsis* (Teige et al., 2004), and both *CsaV3_7G026200* and *CsaV3_7G026220* encode pectinesterase, which catalyze the demethylesterification of cell wall pectins. Demethylesterification influences cell wall rheological properties, which in turn affect plant growth and development, as well as resistance to stress and disease (Anming et al., 2020). It is of note that the closest *Arabidopsis* orthologue of these two pectinesterase genes is a known regulator of plant response to fungal and bacterial infection.

- *CsaV3_7G026140* contained a SNP at position + 15,617,754 bp in the CDS, i.e., a T → C substitution (**Figure 6C**), predicted to lead to an amino acid change from Phe to Leu (**Figure 6C**). Haplotype analysis of this gene among the accessions show that 61 individuals carry the *Hap1* ‘T’ allele, while in contrast, seven accessions have the alternate *Hap2* ‘C’ allele. A significant difference in DI was found between the two contrasting haplotypes (**Figure 6D**). The expression of *CsaV3_7G026140* was significantly up-regulated after TLS infection in ‘CG107’, ‘R28’, and ‘R31’ carrying the *Hap1* allele, while it was down-regulated in ‘CG71’ and ‘CG78’ carrying *Hap2* (**Figure 6E**).
- Three non-synonymous mutations were identified in the CDS region of *CsaV3_7G026180*. C → T substitution at 15,660,836 bp, is predicted to cause a premature termination of the CDS (**Figure 6C**). The *Hap1* and *Hap2* accessions had similar a DI, but both differed significantly from *Hap3* accessions (**Figure 6D**). The expression of *CsaV3_7G026180* was significantly up-regulated at 12 hours post-inoculation in *Hap1* (‘CG107’) and *Hap2* (‘R28’), while it was down-regulated in *Hap3* which included ‘CG72’ and ‘CG78’ (**Figure 6E**).
- The *CsaV3_7G026200* variant contained a SNP at 15,673,237 bp in the CDS, with a G substituted for a C, leading to a Lys → Asn mutation (**Figure 6C**). Haplotype analysis suggested that the DI of both *Hap1* and *Hap2* accessions were significantly different from that of *Hap3* (**Figure 6D**). There was also a significant difference in expression in TLS-resistant vs. sensitive accessions (**Figure 6E**). *CsaV3_7G026140* was significantly up-regulated after TLS infection in *Hap1* (‘CG107’) and *Hap2* (‘R16’ and ‘R31’) accessions at 12 hours compared with those carrying the *Hap3* (‘CG72’ and ‘CG78’) allele (**Figure 6E**).
- G → A SNP at + 15,683,072 bp in the CDS of *CsaV3_7G026220* (**Figure 6C**), is predicted to substitute a Val for an Ala (**Figure 6C**). Haplotype analysis showed that the DI of the *Hap1* and *Hap2* genotypes were significantly different from that of *Hap3* (**Figure 6D**). The expression of *CsaV3_7G026220* was significantly up-regulated in ‘CG107’ carrying the *Hap1* allele and ‘R16’ carrying that of *Hap2* at 12 hours after inoculation compared with accessions carrying *Hap3* (‘CG71’ and ‘CG72’) (**Figure 6E**).

## 4 Discussion

### 4.1 Identification of TLS-resistant accessions at the adult stage

Research on the genetic and molecular mechanisms of cucumber TLS resistance is limited, which constrains resources and breeding efforts for developing resistant cucumber varieties. Currently, resistance studies on TLS have focused on the seedling stage, and no genes related to TLS resistance in adult stage cucumbers have been identified. In this study, we evaluated TLS resistance in 130 adult cucumber accessions using DI, which provided a foundation for selecting new TLS-resistant varieties. We identified 32 TLS-resistant accessions, including all Xishuangbanna type. The four cucumber ecotypes exhibited disparate levels of TLS resistance, with the Eurasian type being more susceptible and the Xishuangbanna type showing stronger resistance. Interestingly, the Xishuangbanna type also demonstrated robust resistance to other diseases such as powdery and downy mildew, gummy stem blight, and bacterial soft rot (Liu et al., 2021, 2020; Han et al., 2023; Zhang et al., 2024), suggesting that it may possess diverse disease-resistant genes. Therefore, the Xishuangbanna type could be a valuable resource for future breeding programs aimed at improving disease resistance.

Moreover, by including two seasons within the same year, the comparison of seasonal differences over a shorter time frame helped control the impact of seasonal variations on plants or diseases. Another experiment conducted in a different year helped validate the applicability of the findings from the two trials under different environmental conditions, thereby improving the accuracy and reliability of the results. However, there are several limitations to acknowledge. First, the use of natural field infections introduced uncontrollable variability, potentially affecting the consistency of results across different seasons. Second, this study focused on the adult stage, while future research should also investigate the genetic mechanisms of TLS resistance during the seedling stage. To address these limitations, future studies should employ artificial inoculation experiments to minimize environmental variability. Further investigation can validate the function of these genes using methods such as CRISPR/Cas9 technology.

### 4.2 GWAS analysis for TLS resistance at the adult stage

To obtain a more reliable GWAS results, the DI values in three seasons were then transformed into BLUP values, which can reduce the influence of environmental factors in previous studies (VanRaden, 2008). The BLUP values showed a significantly positive correlation with the raw DI values and used to perform a GWAS analysis with a linear mixed model (Fast-LMM) (Lippert et al., 2011). Three SNP loci *gTLS5.1* and *gTLS5.2* on Chr.5, and, *gTLS7.1* on Chr.7 were detected with a threshold of 5.1, and five genes in these regions were predicted to be potentially associated with TLS resistance. Previous studies on TLS resistance commonly identified resistance loci or genes on chromosome 6 (Wang et al., 2010; Wen et al., 2015; Yang et al., 2012), suggesting that these loci or genes we detected are novel. This is also the first report identifying novel loci or genes associated with TLS resistance in adult cucumber plants using GWAS analysis.

Although repeated signaling loci could be detected in October 2021 and July 2023, the correlation between these two seasons is not high. It is possibly due to the fact that we used natural infestation in the field in this study. The consistency of experimental conditions can be affected by environmental conditions in different seasons and years under natural infection. In order to obtain a more accurate result, it is recommended that an artificial infection was used to characterize the TLS resistance of the accessions.

### 4.3 Candidate genes for TLS resistance

Our GWAS identified five genes that may be associated with disease resistance and stress tolerance (**Supplementary Table S5**), including *CsaV3_5G010580*, *CsaV3_7G026140*, CsaV3_7G026180, *CsaV3_7G026200*, and *CsaV3_7G026220*. At locus *gTLS5.1*, candidate gene *CsaV3_5G010580* was identified.

*CsaV3_5G010580* encodes a E3 ubiquitin ligase, which not only regulates salt tolerance by degrading GIGANTEA in the roots, but also plays a role in resistance to the fungal pathogen (Deng et al., 2017; Ji et al., 2024). Additionally, the *Arabidopsis* orthologue *AT2G38920* is annotated as a SPX domain-containing protein/zinc finger protein-related gene, which may be associated with disease and stress resistance in plants (**Supplementary Table S6**). *CsaV3_7G026140* encodes an R protein complex member, RPM1-interacting protein 4 (RIN4). RIN4 negatively regulates the basal defense response in plants and is a target for multiple bacterial virulence effectors (Yao et al., 2009). *CsaV3_7G026180* gene encodes a HXXXD-type acyl-transferase protein and its ortholog was responsive to various cold regulatory pathways in tea plant leaves (Hao et al., 2018). Both *CsaV3_7G026200* and *CsaV3_7G026220* encode pectinesterases, which were reported to regulate plant biotic and abiotic tolerance by influencing cell wall fluidity (Anming et al., 2020). These two cucumber genes are 67.57% and 66.91% similar to the closest orthologue in *Arabidopsis*, *AT3G14310*, which negatively regulates fungal and bacterial response (https://www.arabidopsis.org). Interestingly, all genes we predicted were negative-regulated TLS resistance. When facing pathogens invasion, plants need to balance disease resistance with growth and development. Negative regulatory mechanisms help support plant growth and enhance pathogen resistance (Huang et al., 2013; Joydeep et al., 2018; Liu et al., 2021a)

## 5 Conclusion

In summary, we systematically evaluated the phenotypic variation in adult stage resistance to TLS across a diverse cucumber accessions collection. Using DI as the evaluation criterion, we identified 32 highly resistant accessions, with the Xishuangbanna ecotype showing superior TLS resistance compared to other ecotypes. GWAS identified three significant loci (*gTLS5.1*, *gTLS5.2*, and *gTLS7.1*) associated with resistance at the adult stage. Functional annotation identified five candidate genes (*CsaV3_5G010580*, *CsaV3_7G026140*, *CsaV3_7G026180*, *CsaV3_7G026200*, and *CsaV3_7G026220*) involved in ubiquitin-mediated protein degradation, cell wall modification, and pathogen effector surveillance and other processes. This study provides elite accessions resources for breeding TLS-resistant cultivars and sheds light on the polygenic regulatory mechanisms underlying disease resistance in adult cucumber, offering novel targets for precision molecular breeding strategies.

## Data Availability

The original contributions presented in the study are included in the article/**Supplementary Material**. Further inquiries can be directed to the corresponding authors.

## References

[B1] Abul-Hayja Z. Williams P. Peterson C. (1978). Inheritance of resistance to anthracnose and target leaf spot in cucumbers. Plant Dis. Rep. 62, 43–45.

[B2] Anming D. Xianfeng T. Dahai Y. Meng W. Angyan R. Zongchang X.. (2020). Erf4 and myb52 transcription factors play antagonistic roles in regulating homogalacturonan de-methylesterification in arabidopsis seed coat mucilage. Plant Cell 33, 381–403. doi: 10.1093/PLCELL/KOAA031 PMC8136884 33709105

[B3] Deng F. Guo T. M. L. Scaglione S. Antico C. Jing T.. (2017). Expression and regulation of atl9, an e3 ubiquitin ligase involved in plant defense. PloS One 12, e0188458. doi: 10.1371/journal.pone.0188458 29161311 PMC5697834

[B4] Han J. Dong S. Shi Y. Dai Z. Miao H. Li B.. (2023). Genome-wide association study reveals candidate genes for gummy stem blight resistance in cucumber. Hortic. Plant J. 9, 261–272. doi: 10.1016/j.hpj.2022.06.004

[B5] Hao X. Wang B. Wang L. Zeng J. Yang Y. Wang X. (2018). Comprehensive transcriptome analysis reveals common and specific genes and pathways involved in cold acclimation and cold stress in tea plant leaves. Scientia Hortic. 240, 354–368. doi: 10.1016/j.scienta.2018.06.008

[B6] Huang S. Li R. Zhang Z. Li L. Gu X. Fan W.. (2009). The genome of the cucumber, *cucumis sativus* L. Nat. Genet. 41, 1275–1281. doi: 10.1038/ng.475 19881527

[B7] Huang Y. Chen X. Liu Y. Roth C. Copeland C. McFarlane H.. (2013). Mitochondrial AtPAM16 is required for plant survival and the negative regulation of plant immunity. Nat. Commun. 4, 2558. doi: 10.1038/ncomms3558 24153405

[B8] Jana J. Jiban K. (2010). Validation of reference genes as internal control for studying viral infections in cereals by quantitative real-time rt-pcr. BMC Plant Biol. 10, 146. doi: 10.1186/1471-2229-10-146 20630112 PMC3095291

[B9] Ji M. Khakurel D. Hwang J. Nguyen C. Nam B. Shin G.. (2024). The e3 ubiquitin ligase cop1 regulates salt tolerance via gigantea degradation in roots. Plant Cell Environ. 47, 3241–3252. doi: 10.1111/PCE.14946 38741272

[B10] Jiang L. Zheng Z. Fang H. Yang J. (2021). A generalized linear mixed model association tool for biobank-scale data. Nat. Genet. 53, 1616–1621. doi: 10.1038/S41588-021-00954-4 34737426

[B11] Joydeep C. Prithwi G. Sampa D. (2018). Autoimmunity in plants. Planta 248, 751–767. doi: 10.1007/s00425-018-2956-0 30046903

[B12] Kan L. Li B. Ji M. Zhang Z. Shi Y. (2007). Screening agents for controlling target leaf spots on cucumbers. China Vegetables 04), 22–24.

[B13] Li C. Dong S. Beckles D. M. Liu X. Guan J. Gu X.. (2023). GWAS reveals novel loci and identifies a pentatricopeptide repeat-containing protein (CsPPR) that improves low temperature germination in cucumber. Front. Plant Sci. 14. doi: 10.3389/fpls.2023.1116214 PMC10208356 37235012

[B14] Li Y. (2010). Target spot disease detection and control in cucumber. Modern Agric. Sci. Technol. 11), 177.

[B15] Li Y. Wang Y. Wu X. Wang J. Wu X. Wang B.. (2021). Novel genomic regions of fusarium wilt resistance in bottle gourd [Lagenaria siceraria (Mol.) Standl.] discovered in genome-wide association study. Front. Plant Sci. 12, 650157–650157. doi: 10.3389/FPLS.2021.650157 34025697 PMC8137845

[B16] Li B. Zhao Y. Yu S. Chai A. Gao W. (2008). Dr. Li baoju’s disease handbook (vi) cucumber leaf spot disease of cucumis sativus in Qingxian, Hebei in the fall of 2008. China Vegetables 11, 51–52. doi: 10.19928/j.cnki.1000-6346.2008.11.022

[B17] Lippert C. Listgarten J. Liu Y. Kadie C. M. Davidson R. I. Heckerman D. (2011). FaST linear mixed models for genome-wide association studies. Nat. Methods 8, 833–835. doi: 10.1038/nmeth.1681 21892150

[B18] Liu X. Gu X. Lu H. Liu P. Miao H. Bai Y.. (2021a). Identification of novel loci and candidate genes for resistance to powdery mildew in a resequenced cucumber germplasm. Genes 12, 584. doi: 10.3390/genes12040584 33923788 PMC8072792

[B19] Liu H. Li H. Hu Y. Yang Y. Zhang W. He M.. (2021). EDS1-interacting J protein 1 is an essential negative regulator of plant innate immunity in *Arabidopsis* . Plant Cell 33, 153–171. doi: 10.1093/PLCELL/KOAA007 33751092 PMC8136891

[B20] Liu X. Lu H. Liu P. Miao H. Bai Y. Gu X.. (2020). Identification of novel loci and candidate genes for cucumber downy mildew resistance using GWAS. Plants 9, 1659. doi: 10.3390/plants9121659 33260843 PMC7768435

[B21] Liu H. Yan J. (2019). Crop genome-wide association study: A harvest of biological relevance. Plant J. 97, 8–18. doi: 10.1111/tpj.14139 30368955

[B22] Ma Z. Zhang Y. Wu L. Zhang G. Sun Z. Li Z.. (2021). High-quality genome assembly and resequencing of modern cotton cultivars provide resources for crop improvement. Nat. Genet. 53, 1385–1391. doi: 10.1038/S41588-021-00910-2 34373642 PMC8423627

[B23] Manickam R. Raja C. Chinnasamy S. (2023). An analysis on solar photovoltaic technology using IBM SPSS statistics. J. Electronic Automation Eng. 2, 38–46. doi: 10.46632/jeae/2/1/5

[B24] Purcell S. Neale B. Todd-Brown K. Thomas L. Ferreira M. Bender D. (2007). Plink: A tool set for whole-genome association and population-based linkage analyses. Am. J. Hum. Genet. 81, 559–575. doi: 10.1086/519795 17701901 PMC1950838

[B25] Qi J. Liu X. Shen D. Miao H. Xie B. Li X.. (2013). A genomic variation map provides insights into the genetic basis of cucumber domestication and diversity. Nat. Genet. 45, 1510–1515. doi: 10.1038/ng.2801 24141363

[B26] Shin J.-H. Blay S. McNeney B. Graham J. (2006). LDheatmap: an R function for graphical display of pairwise linkage disequilibria between single nucleotide polymorphisms. J. Stat. Software 16, 1–9. doi: 10.18637/jss.v016.c03

[B27] Team, R.C (2014). “R: A language and environment for statistical computing,” in MSOR connections.

[B28] Teige M. Scheikl E. Eulgem T. Dóczi R. Ichimura K. Shinozaki K.. (2004). The MKK2 pathway mediates cold and salt stress signaling in Arabidopsis. Mol. Cell 15, 141–152. doi: 10.1016/j.molcel.2004.06.023 15225555

[B29] Turesson G. (1922). The genotypical response of the plant species to the habitat. Hereditas 3, 211–350. doi: 10.1111/j.1601-5223.tb02734.x

[B30] VanRaden P. (2008). Efficient methods to compute genomic predictions. J. Dairy Sci. 91, 4414–4423. doi: 10.3168/jds.2007-0980 18946147

[B31] Wang H. Li S. Guan W. (2010). Identification of sources of resistance to cucumber target leaf spot and genetic analysis of resistance. Chin. Cucurbits Vegetables 23, 24–25. doi: 10.16861/j.cnki.zggc.2010.01.009

[B32] Wen C. Mao A. Dong C. Liu H. Yu S. Guo Y.. (2015). Fine genetic mapping of target leaf spot resistance gene cca-3 in cucumber, *cucumis sativus* L. TAG Theor. Appl. Genet. Theoretische und angewandte Genetik 128, 2495–2506. doi: 10.1007/s00122-015-2604-z 26385372

[B33] Wu X. Wang Y. Wu X. Xu P. Wu B. G. Lu Z. F.. (2020). Genome-wide association analysis of powdery mildew resistance in gourd (lagenaria siceraria). Molplantbreed 18, 759–764. doi: 10.3969/j.issn.1004-1524.20221361

[B34] Xie Q. Liu P. Shi L. Miao H. Bo K. Wang Y.. (2018). Combined fine mapping, genetic diversity, and transcriptome profiling reveals that the auxin transporter gene ns plays an important role in cucumber fruit spine development. TAG Theor. Appl. Genet. Theoretische und angewandte Genetik 131, 1239–1252. doi: 10.1007/s00122-018-3074-x 29492617

[B35] Yang S. Gu X. Zhang S. Li B. (2012). Research progress on cucumber target leaf spot (*Corynespora cassiicola*). China Vegetables 04), 1–9. doi: 10.19928/j.cnki.1000-6346.2012.04.001

[B36] Yao L. Caldwell K. S. Tadeusz W. Wroblewski T. Wright M. E. Michelmore R. W. (2009). Proteolysis of a negative regulator of innate immunity is dependent on resistance genes in tomato and nicotiana benthamiana and induced by multiple bacterial effectors. Plant Cell 21, 2458–2472. doi: 10.1105/tpc.107.056044 19671880 PMC2751963

[B37] Yu S. Wang M. Tian F. Zhao W. Bian Q. Li B. (2014). Research on controlling and resisting cucumber clubroot leaf spot disease. Pesticide J. 53, 7–11. doi: 10.16820/j.cnki.1006-0413.2014.01.003

[B38] Zhang Y. Dong S. Guan J. Liu X. Xie X. Albornoz K.. (2024). Genome-wide association study identifies candidate genes for bacterial soft rot resistance in cucumber seedlings. Hortic. Plant J. 11 (3), 1152–1165. doi: 10.1016/j.hpj.2024.02.006

[B39] Zhou M. Coruh C. Xu G. Martins L. Bourbousse C. Lambolez A.. (2022). The CLASSY family controls tissue-specific DNA methylation patterns in *Arabidopsis* . Nat. Commun. 1), 244–244. doi: 10.1038/S41467-021-27690-X PMC8752594 35017514

[B40] Zou Q. Fu J. Zhu Y. Fang D. (2002). Identification and biological characterization of cucumber brown spot pathogenic bacteria. J. Shenyang Univ. Agric. 04), 258–261.

