# Supplementary material for: A genome-wide association study identifies candidate genes for target leaf spot disease resistance in adult cucumber (Cucumis sativus L.)
Source: Front Plant Sci. 2025 Jun 6;16:1542274. doi: 10.3389/fpls.2025.1542274 (PMC12179077; doi:10.3389/fpls.2025.1542274)
Supplement: Supplementary file 1 [file Supplementaryfile1.docx]

***Supplementary Material***

**A genome-wide association study identifies candidate genes for target leaf spot disease resistance in adult cucumber (*Cucumis sativus* L.)**

^
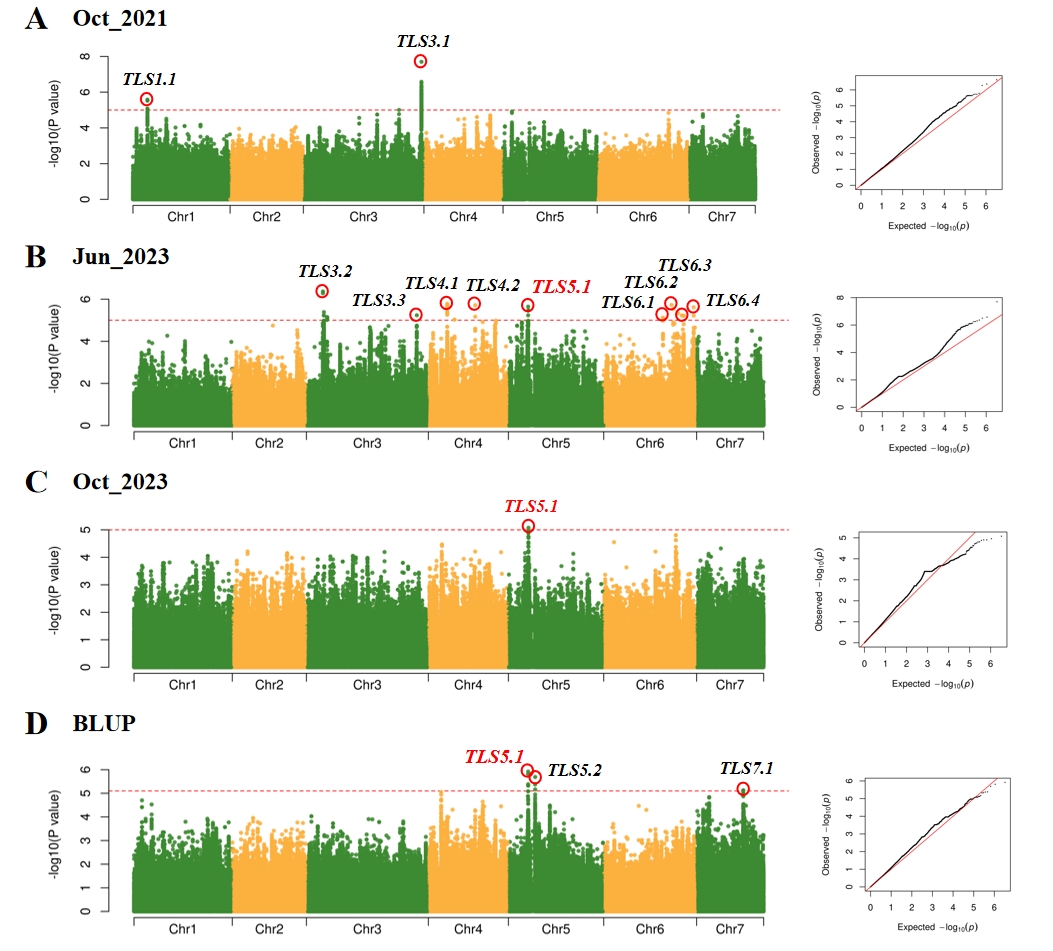
^

Fig. S1: GWAS Manhattan plot and Q-Q plot of TLS resistance in Oct_2021, Jun_2023, Oct_2023 and BLUP.

The red horizontal line represents a significance threshold of 5.1 (-log10(*p*)>5.1), and the strongest peak is marked with a red circle.Reddish colored loci indicate duplicate detected loci.


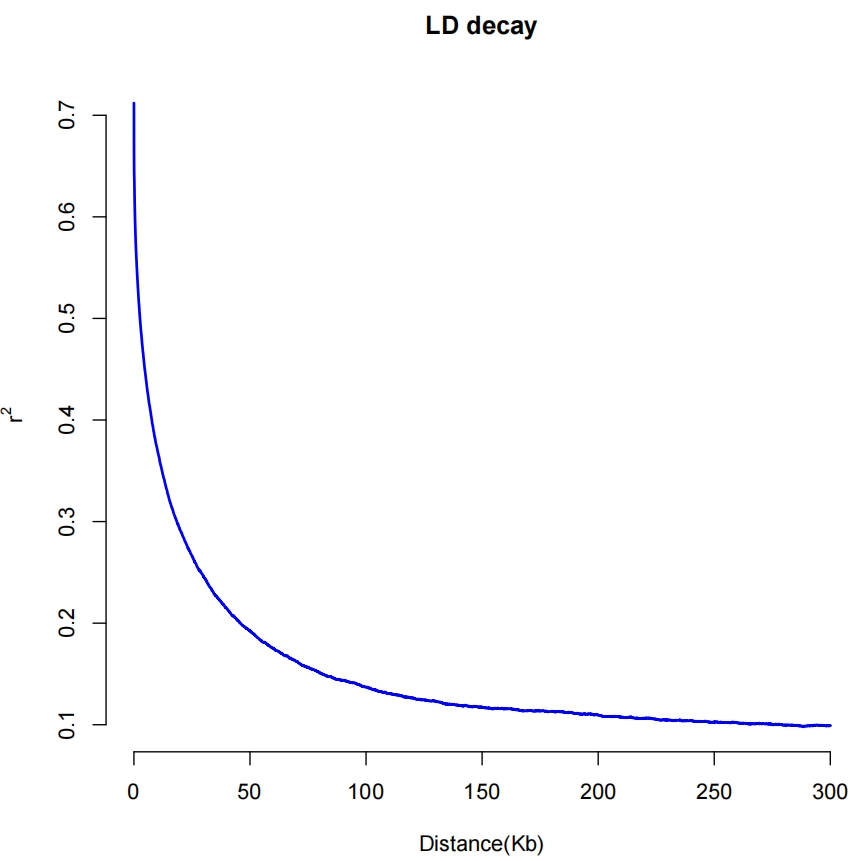


Fig. S2: LD decay pattern of 130 core accessions across genetic distances.The x-axis represents the genetic distance in kilobases (Kb), and the y-axis shows the LD coefficient (r^2^).


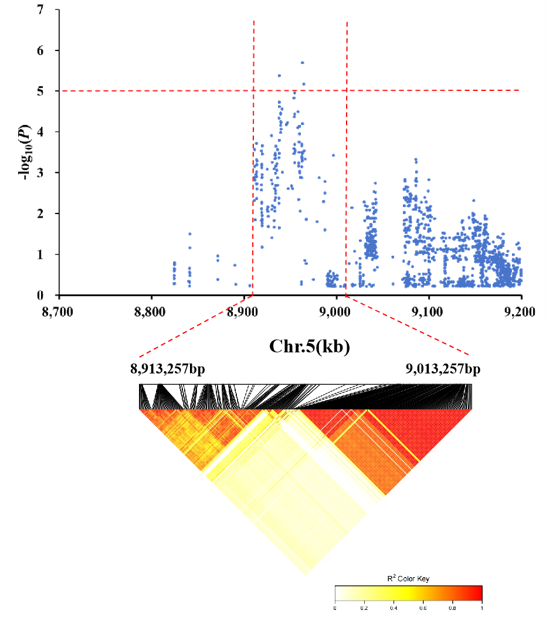


Fig. S3: Local Manhattan plot (above) and LD heatmap (below) surrounding *gTLS5.2*. The horizontal red dashed line indicates the significance threshold (-log_10_(*P*) > 5.1). The vertical red dashed lines indicate the candidate region (100 kb).
