# Supplementary material for: A genome-wide association study identifies candidate genes for target leaf spot disease resistance in adult cucumber (Cucumis sativus L.)
Source: Front Plant Sci. 2025 Jun 6;16:1542274. doi: 10.3389/fpls.2025.1542274 (PMC12179077; doi:10.3389/fpls.2025.1542274)
Supplement: Supplementary file 2 [file Table1.docx]

Table S1 Target leaf spot disease resistance data of 130 cucumber accessions over three seasons

| **Code** | **Oct_2021** | **Jun_2023** | **Oct_2023** | **BLUP_value** |
| --- | --- | --- | --- | --- |
| CG1 | 24.00 | 46.67 | 33.33 | 35.04 |
| CG2 | NA | 22.22 | 44.44 | 33.38 |
| CG3 | 18.00 | 35.56 | 55.56 | 36.07 |
| CG4 | NA | 33.89 | 66.67 | 41.86 |
| CG5 | NA | 42.22 | NA | 38.76 |
| CG6 | 20.00 | 44.44 | 33.33 | 33.80 |
| R57 | 20.00 | 20.00 | 33.33 | 28.90 |
| R58 | 16.00 | 54.44 | 33.33 | 35.00 |
| CG9 | 44.00 | 31.11 | 66.67 | 42.61 |
| CG10 | 18.00 | 47.22 | 33.33 | 33.95 |
| CG11 | NA | 60.00 | 55.56 | 45.62 |
| CG12 | 56.00 | 44.44 | 77.78 | 49.90 |
| CG13 | 36.00 | 77.78 | 77.78 | 52.57 |
| CG14 | 34.00 | 25.00 | 66.67 | 39.38 |
| CG15 | 32.00 | 31.85 | 55.56 | 38.13 |
| CG16 | 30.00 | 31.11 | 55.56 | 37.58 |
| CG17 | NA | 33.33 | 55.56 | 38.94 |
| CG19 | 24.00 | 30.00 | 33.33 | 31.71 |
| CG21 | 50.00 | 29.44 | 55.56 | 41.25 |
| CG23 | 16.00 | 30.56 | 44.44 | 32.44 |
| CG25 | 48.00 | 50.00 | 55.56 | 44.96 |
| R60 | 56.00 | 50.00 | NA | 46.11 |
| CG27 | NA | 33.33 | NA | 35.80 |
| CG28 | 12.00 | 21.11 | 11.11 | 23.08 |
| CG29 | 24.00 | 33.89 | 33.33 | 32.48 |
| CG30 | 18.00 | 27.78 | NA | 31.04 |
| CG31 | 56.00 | 50.00 | 44.44 | 44.34 |
| CG32 | 28.00 | 44.44 | 66.67 | 42.07 |
| R47 | 42.00 | 26.67 | 55.56 | 39.09 |
| CG35 | 38.00 | 29.44 | 33.33 | 34.40 |
| CG36 | 24.00 | 15.74 | 44.44 | 31.08 |
| R59 | 56.00 | 91.67 | 55.56 | 54.91 |
| R46 | NA | 25.00 | 66.67 | 39.64 |
| CG40 | NA | 18.52 | 33.33 | 29.67 |
| CG43 | NA | 42.59 | 44.44 | 38.48 |
| CG44 | 16.00 | 27.78 | 33.33 | 29.66 |
| CG45 | 38.00 | 28.33 | 33.33 | 34.17 |
| CG49 | 36.00 | 38.89 | 33.33 | 35.89 |
| CG50 | 44.00 | NA | 44.44 | 39.21 |
| CG51 | 58.00 | 33.89 | 55.56 | 43.74 |
| R146 | NA | 11.11 | NA | 28.38 |
| R61 | NA | 4.44 | NA | 26.15 |
| R62 | 24.00 | 50.00 | 33.33 | 35.71 |
| R63 | 18.00 | 38.89 | 55.56 | 36.73 |
| R50 | 16.00 | 33.33 | 44.44 | 33.00 |
| CG59 | 20.00 | 25.00 | 33.33 | 29.90 |
| CG60 | 36.00 | 34.81 | 44.44 | 37.30 |
| R64 | 22.00 | 25.56 | 33.33 | 30.42 |
| R65 | 20.00 | 36.11 | 55.56 | 36.58 |
| CG64 | 8.00 | 17.59 | 33.33 | 26.02 |
| CG66 | NA | 22.22 | NA | 32.09 |
| CG69 | 32.00 | 16.67 | 44.44 | 32.86 |
| CG70 | 20.00 | 11.11 | 11.11 | 22.67 |
| CG71 | 14.00 | 22.22 | 22.22 | 25.92 |
| CG72 | 14.00 | 27.78 | 33.33 | 29.26 |
| CG77 | NA | 33.33 | NA | 35.80 |
| CG78 | 24.00 | 28.15 | 22.22 | 29.11 |
| CG84 | 20.00 | 4.63 | 11.11 | 21.38 |
| CG85 | NA | 33.33 | 55.56 | 38.94 |
| CG86 | 8.00 | 20.37 | 11.11 | 22.13 |
| CG87 | 28.00 | 31.11 | 33.33 | 32.73 |
| CG88 | NA | 22.22 | 44.44 | 33.38 |
| CG89 | 20.00 | 22.22 | 44.44 | 31.57 |
| R66 | 28.00 | 35.56 | 33.33 | 33.62 |
| CG91 | 16.00 | 33.89 | 44.44 | 33.11 |
| CG92 | 42.00 | 23.61 | 44.44 | 36.25 |
| R67 | 24.00 | 48.89 | 55.56 | 39.94 |
| CG94 | 38.00 | 22.22 | 33.33 | 32.95 |
| R68 | 34.00 | 27.22 | 55.56 | 37.60 |
| CG96 | 20.00 | 24.44 | 44.44 | 32.02 |
| R69 | 36.00 | 33.33 | 44.44 | 37.00 |
| CG98 | 20.00 | 14.81 | 22.22 | 25.64 |
| CG99 | 26.00 | 41.67 | 33.33 | 34.44 |
| CG100 | 12.00 | 18.52 | 22.22 | 24.78 |
| R72 | 44.00 | 17.78 | 55.56 | 37.71 |
| R70 | 30.00 | 19.44 | NA | 31.96 |
| CG104 | 6.00 | 33.33 | 22.22 | 26.55 |
| R71 | 38.00 | 25.00 | 44.44 | 35.73 |
| CG106 | 32.00 | 27.41 | 33.33 | 32.79 |
| CG107 | 44.00 | 30.56 | 55.56 | 40.27 |
| CG108 | 48.00 | 22.22 | 55.56 | 39.40 |
| CG109 | 36.00 | 38.89 | 55.56 | 40.34 |
| CG110 | NA | 28.89 | 44.44 | 35.05 |
| CG112 | 28.00 | 44.44 | 55.56 | 39.85 |
| CG113 | 12.00 | 41.11 | 33.33 | 31.53 |
| CG114 | 44.00 | 27.78 | 44.44 | 37.49 |
| CG117 | 28.00 | 38.89 | 33.33 | 34.29 |
| CG118 | 24.00 | 40.00 | 33.33 | 33.71 |
| CG120 | NA | 18.52 | NA | 30.85 |
| CG201 | NA | 37.78 | 33.33 | 34.49 |
| R4 | 30.00 | 55.56 | NA | 41.00 |
| R8 | NA | 42.59 | NA | 38.89 |
| R13 | 40.00 | 16.11 | 55.56 | 36.58 |
| R16 | 30.00 | 31.48 | 33.33 | 33.20 |
| R19 | 28.00 | 43.89 | 55.56 | 39.74 |
| R21 | 52.00 | 29.63 | 33.33 | 37.24 |
| R28 | NA | 55.56 | 55.56 | 44.51 |
| R31 | 38.00 | 30.56 | 33.33 | 34.62 |
| R34 | 32.00 | 44.44 | 55.56 | 40.65 |
| R39 | NA | 30.37 | 55.56 | 38.20 |
| R44 | 36.00 | 41.67 | 55.56 | 40.89 |
| R48 | 20.00 | 31.11 | 44.44 | 33.35 |
| R51 | 32.00 | 22.22 | 33.33 | 31.75 |
| R54 | 38.00 | 23.33 | 33.33 | 33.17 |
| R74 | 32.00 | 26.67 | 33.33 | 32.64 |
| R75 | 44.00 | 22.22 | 55.56 | 38.60 |
| R77 | 12.00 | 31.11 | 33.33 | 29.53 |
| R83 | 22.00 | 24.44 | 77.78 | 39.09 |
| R84 | 38.00 | 42.78 | 55.56 | 41.52 |
| R87 | NA | 50.00 | 44.44 | 40.33 |
| R89 | 32.00 | 39.44 | 33.33 | 35.20 |
| R90 | 44.00 | 30.56 | 33.33 | 35.82 |
| R92 | NA | 40.00 | 33.33 | 35.05 |
| R93 | 50.00 | 33.33 | 33.33 | 37.58 |
| R95 | 22.00 | 50.00 | 33.33 | 35.31 |
| R97 | 24.00 | 40.74 | 33.33 | 33.86 |
| R99 | 64.00 | 27.22 | 55.56 | 43.61 |
| R100 | 48.00 | NA | 55.56 | 42.99 |
| R104 | NA | 15.28 | NA | 29.77 |
| R107 | NA | 25.00 | 55.56 | 36.86 |
| R110 | 42.00 | 21.11 | 33.33 | 33.53 |
| R117 | 50.00 | 31.11 | 44.44 | 39.36 |
| R120 | 36.00 | 20.00 | 55.56 | 36.56 |
| R122 | 56.00 | 28.89 | 55.56 | 42.34 |
| R124 | NA | 23.33 | 33.33 | 30.88 |
| R127 | 40.00 | NA | 33.33 | 35.43 |
| R137 | NA | 52.59 | 44.44 | 40.98 |
| R148 | NA | 53.33 | 55.56 | 43.95 |
| R163 | 6.00 | 31.48 | 22.22 | 26.17 |
| R164 | 32.00 | 34.44 | 33.33 | 34.20 |

Table S2 Ecotype information of cucumber accessions used for TLS resistance characterization

| **Code** | **Ecotype** |
| --- | --- |
| CG1 | Eurasian |
| CG2 | Indian |
| CG3 | East Asian |
| CG4 | East Asian |
| CG5 | Eurasian |
| CG6 | East Asian |
| CG9 | Eurasian |
| CG10 | Eurasian |
| CG11 | Eurasian |
| CG12 | Indian |
| CG13 | Eurasian |
| CG14 | Indian |
| CG15 | Indian |
| CG16 | Indian |
| CG17 | Indian |
| CG19 | Indian |
| CG21 | Indian |
| CG23 | Indian |
| CG25 | East Asian |
| R60 | East Asian |
| CG27 | East Asian |
| CG28 | East Asian |
| CG29 | East Asian |
| CG30 | East Asian |
| CG31 | East Asian |
| CG32 | Eurasian |
| R47 | Eurasian |
| CG35 | Eurasian |
| CG36 | Eurasian |
| R59 | Eurasian |
| R46 | Eurasian |
| CG40 | Eurasian |
| CG43 | Eurasian |
| CG45 | Eurasian |
| CG49 | Indian |
| CG50 | Indian |
| CG51 | Indian |
| R146 | Eurasian |
| R62 | East Asian |
| R63 | East Asian |
| R50 | East Asian |
| CG59 | East Asian |
| CG60 | East Asian |
| R64 | East Asian |
| CG64 | Indian |
| CG66 | Xishuangbanna |
| CG69 | Xishuangbanna |
| CG70 | Xishuangbanna |
| CG71 | Xishuangbanna |
| CG72 | Xishuangbanna |
| CG77 | Xishuangbanna |
| CG78 | Xishuangbanna |
| CG84 | Xishuangbanna |
| CG85 | Xishuangbanna |
| CG86 | Indian |
| CG87 | Indian |
| CG88 | Indian |
| CG89 | East Asian |
| R66 | East Asian |
| CG91 | East Asian |
| CG92 | East Asian |
| R67 | East Asian |
| CG94 | East Asian |
| R68 | East Asian |
| CG96 | East Asian |
| R69 | East Asian |
| CG98 | East Asian |
| CG99 | East Asian |
| CG100 | East Asian |
| R72 | East Asian |
| R70 | East Asian |
| CG104 | East Asian |
| R71 | East Asian |
| CG106 | East Asian |
| CG107 | East Asian |
| CG108 | East Asian |
| CG109 | Eurasian |
| CG110 | Eurasian |
| CG112 | Eurasian |
| CG113 | Eurasian |
| CG114 | Eurasian |
| CG117 | Indian |
| CG118 | Indian |
| CG120 | East Asian |
| CG201 | East Asian |
| R4 | Eurasian |
| R8 | Eurasian |
| R13 | East Asian |
| R16 | East Asian |
| R19 | East Asian |
| R21 | East Asian |
| R28 | East Asian |
| R31 | Eurasian |
| R34 | East Asian |
| R39 | Eurasian |
| R44 | Eurasian |
| R48 | Eurasian |
| R51 | East Asian |
| R54 | East Asian |
| R74 | East Asian |
| R75 | Eurasian |
| R77 | Eurasian |
| R83 | Eurasian |
| R84 | Eurasian |
| R87 | Eurasian |
| R89 | East Asian |
| R90 | East Asian |
| R92 | East Asian |
| R93 | East Asian |
| R95 | East Asian |
| R97 | East Asian |
| R99 | East Asian |
| R100 | East Asian |
| R104 | East Asian |
| R107 | East Asian |
| R110 | East Asian |
| R117 | East Asian |
| R120 | East Asian |
| R122 | East Asian |
| R124 | East Asian |
| R163 | East Asian |
| R164 | East Asian |
| R57 | NA |
| R58 | NA |
| CG44 | NA |
| R61 | NA |
| R65 | NA |
| R127 | NA |
| R137 | NA |
| R148 | NA |

Table S3 Primers used for the real time fluorescence quantitative PCR

| **Gene name** | **Forward primer** | **Reverse primer** |
| --- | --- | --- |
| *CsaV3_5G010580* | CCTTCAGAATTCTCCACTCACT | TTTCAACACTCTAGCTCGTTCT |
| *CsaV3_7G026140* | GAGAGACAGTTCGATGACAGAG | GGAGGGGAATTGTTTTTCGTTT |
| *CsaV3_7G026180* | GCACATTCATGATAAAGTTCAACCC | TTCTAGTAGCGTTAATGGTGGG |
| *CsaV3_7G026200* | GACTTGAATCAAAACACGGGAA | TTATGTCACCATTCCATGTGGA |
| *CsaV3_7G026220* | GTGTCATAGAAACCTCATGTGC | CCATAGCAACACTCAATGACAC |
| *Actin1* | TCCACGAGACTACCTACAACTC | GCTCATACGGTCAGCGAT |

Table S4 Statistics of disease index in core accessions

| **Environment** | **Max** | **Min** | **Mean** | **STD** | **Skewness** | **Kurtosis** | **CV (%)** |
| --- | --- | --- | --- | --- | --- | --- | --- |
| Oct_2021 | 64.00 | 6.00 | 24.28 | 17.33 | 0.08 | -0.92 | 71.37 |
| Jun_2023 | 91.67 | 4.44 | 31.86 | 13.67 | 0.76 | 2.87 | 42.91 |
| Oct_2023 | 77.78 | 11.11 | 38.80 | 18.52 | -0.53 | 0.03 | 47.73 |
| BLUP | 54.91 | 21.38 | 35.61 | 5.79 | 0.21 | 0.90 | 16.25 |

Table S5 Signal locus and genes information detected by GWAS analysis

| **GWAS loci** | **Gene name** | **Physical location** |
| --- | --- | --- |
| *gTLS5.1* | *CsaV3_5G010570* | 6559015-6566713 |
|  | *CsaV3_5G010580* | 6577661-6577661 |
|  | *CsaV3_5G010590* | 6584271-6589990 |
|  | *CsaV3_5G010600* | 6592954-6601046 |
|  | *CsaV3_5G010610* | 6594379-6597605 |
|  | *CsaV3_5G010620* | 6603299-6606603 |
|  | *CsaV3_5G010630* | 6611319-6611498 |
|  | *CsaV3_5G010640* | 6612624-6614285 |
|  | *CsaV3_5G010650* | 6615671-6617504 |
|  | *CsaV3_5G010660* | 6623100-6627126 |
|  | *CsaV3_5G010670* | 6635734-6642344 |
|  | *CsaV3_5G010680* | 6638754-6642402 |
|  | *CsaV3_5G010690* | 6646744-6653284 |
| *gTLS5.2* | *CsaV3_5G012840* | 8923929-8925192 |
|  | *CsaV3_5G012850* | 8940728-8960168 |
|  | *CsaV3_5G012860* | 8967816-8973248 |
| *gTLS7.1* | *CsaV3_7G026140* | 15616593-15618959 |
|  | *CsaV3_7G026150* | 15621705-15624445 |
|  | *CsaV3_7G026160* | 15625414-15631096 |
|  | *CsaV3_7G026170* | 15646962-15652297 |
|  | *CsaV3_7G026180* | 15658075-15661246 |
|  | *CsaV3_7G026190* | 15667092-15667310 |
|  | *CsaV3_7G026200* | 15672436-15679758 |
|  | *CsaV3_7G026210* | 15680275-15681974 |
|  | *CsaV3_7G026220* | 15682775-15687248 |

Table S6 Annotation of candidate genes within signal locus detected by GWAS

| **Gene name** | **Gene annotation** | **Homologous genes of Arabidopsis thaliana** | **Function annotation** |
| --- | --- | --- | --- |
| *CsaV3_5G010580* | RING-type E3 ubiquitin ligase | *AT2G38920* | SPX (SYG1/Pho81/XPR1) domain-containing protein / zinc finger (C3HC4-type RING finger) protein-related |
| *CsaV3_7G026140* | RPM1-interacting protein 4 | *AT3G25070* | Encodes an R protein complex member targeted by type III pili effector proteins from bacterial pathogens, guarded by the RPM1 and RPS2 proteins. |
| *CsaV3_7G026180* | HXXXD-type acyl-transferase family protein | *AT3G29590* | HXXXD-type acyl-transferase family protein |
| *CsaV3_7G026200* | Pectinesterase | *AT3G14310* | pectin methylesterase 3 |
| *CsaV3_7G026220* | Pectinesterase | *AT3G14310* | pectin methylesterase 3 |

Table S7 Cluster result of cucumber accessions for TLS resistance at adult stage

| **TLS resistance groups** | **Groups** | **Code** |
| --- | --- | --- |
| very highly resistant group | I | CG28, CG70, CG84, CG86, CG100, CG104, CG98, R163, R61, CG64, CG71 |
| highly resistant group | II | R146, R57, CG72, CG78, CG59, R77, R104, CG40, CG44, R64,CG120,R124,CG30,CG36,CG66,CG96,R70,CG113,CG89,CG19,R51 |
| medium resistant group | III | CG94,R50,CG91,R16,R54,CG23,CG29,R74,CG69,CG106,CG87,CG117,CG45,R164,R31,CG201,CG35,CG99,CG10,CG118,CG6,R97,R110,R66,R48,CG2,CG88 |
| highly sensitive group | IV | CG108,CG14,R117,CG112,R67,R19,R46,CG15,R39,CG43,R75,CG50,R47,R83,CG5,R8,CG17,CG85,R120,R13,R65,R69,R107,R63,CG60,R21,R72,CG114,R68,CG16,R93,R127,R89,R95,R58,CG1,CG110,R92,CG3,CG92,R62,R71,CG49,R90,CG27,CG77 |
| very highly sensitive group | V | CG12,CG13,R59,CG11,R60,R148,CG51,R99,CG25,CG31,R28,CG107,CG109,R87,R34,R44,R137,R4,R100,CG9,R122,CG32,CG4,CG21,R84 |
